# Supplementary material for: Cardioprotective Effect of Novel Matrix Metalloproteinase Inhibitors
Source: Int J Mol Sci. 2020 Sep 23;21(19):6990. doi: 10.3390/ijms21196990 (PMC7582346; doi:10.3390/ijms21196990)
Supplement: Supplementary file 1 [file ijms-21-06990-s001.pdf]

Supplementary material
of
"Cardioprotective effect of novel matrix metalloproteinase inhibitors"

by Kamilla Gömöri, Tamara Szabados, Éva Kenyeres, Judit Pipis, Imre Földesi, Andrea Siska, György Dormán, Péter Ferdinandy, Anikó Görbe and Péter Bencsik

2020

Normocholesterolemic animal model

Mortality

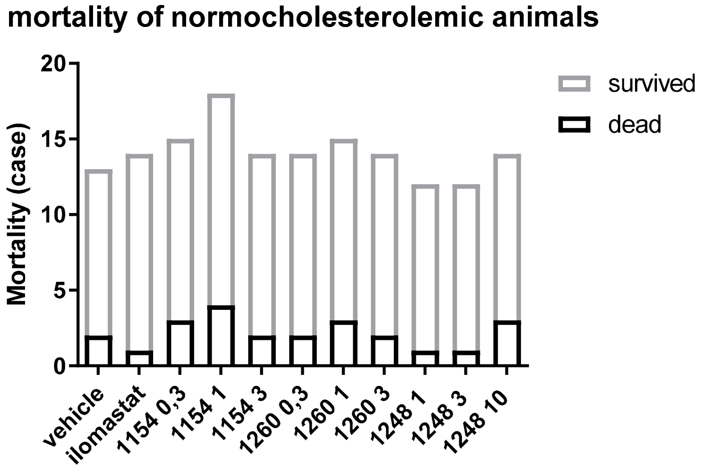

Supplementary Figure S1.All-cause mortality of MMPI-1154, MMPI-1260 and MMPI-1248 test groups. Khi-square test, n=11-17, *p<0.05, no significant difference between groups.

Infarct size measurement

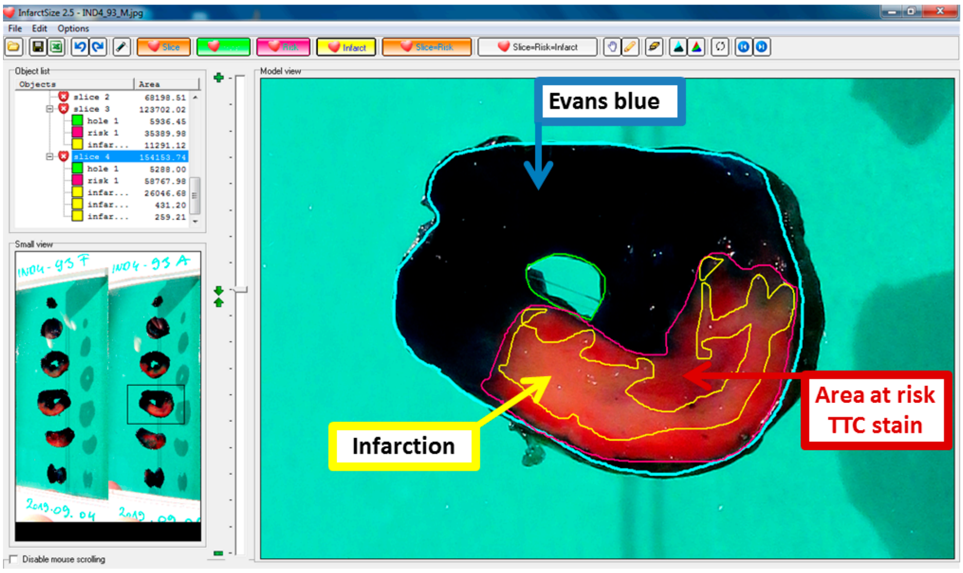

Supplementary Figure S2. Infarct size measurement of rat heart slices after Evans blue and 2,3,5-Triphenyl-tetrazolium chloride (TTC) double staining. White: infarcted region, red: area at risk, blue: non-ischemic region.

Area at risk

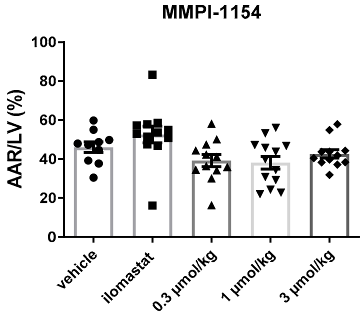

Supplementary Figure S3.A) Area at risk of MMPI-1154 test groups. One-way ANOVA, Fisher LSD *post hoc* test, n=10-12, *p<0.05.

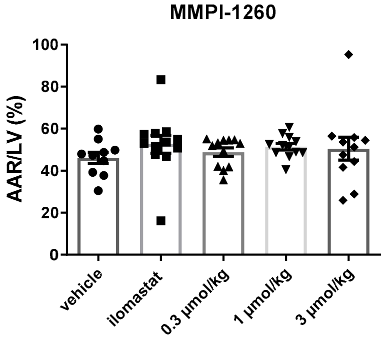

Supplementary Figure S3.B) Area at risk of MMPI-1260 test groups. One-way ANOVA, Fisher LSD *post hoc* test, n=10-12, *p<0.05.

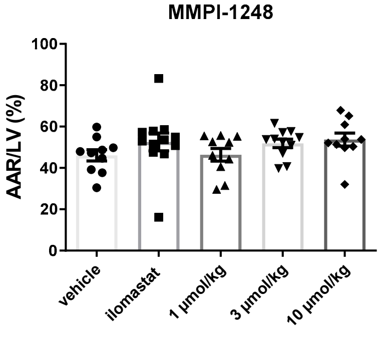

Supplementary Figure S3.C) Area at risk of MMPI-1248 test groups. One-way ANOVA, Fisher LSD *post hoc* test, n=10-12, *p<0.05.

Arrythmia

Incidence of severe arrhythmias

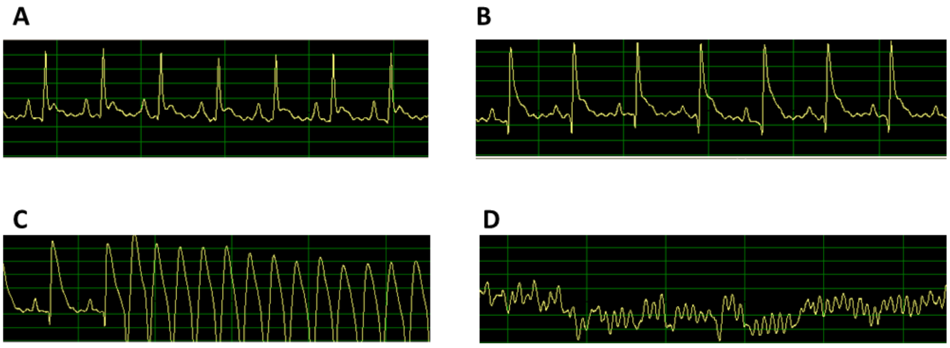

Supplementary Figure S4. Representative recordings of rat ECG. A) normal baseline B) ST-elevation and negative Q wave of ischemic period C) ventricular tachycardia (VT) D) ventricular fibrillation (VF).

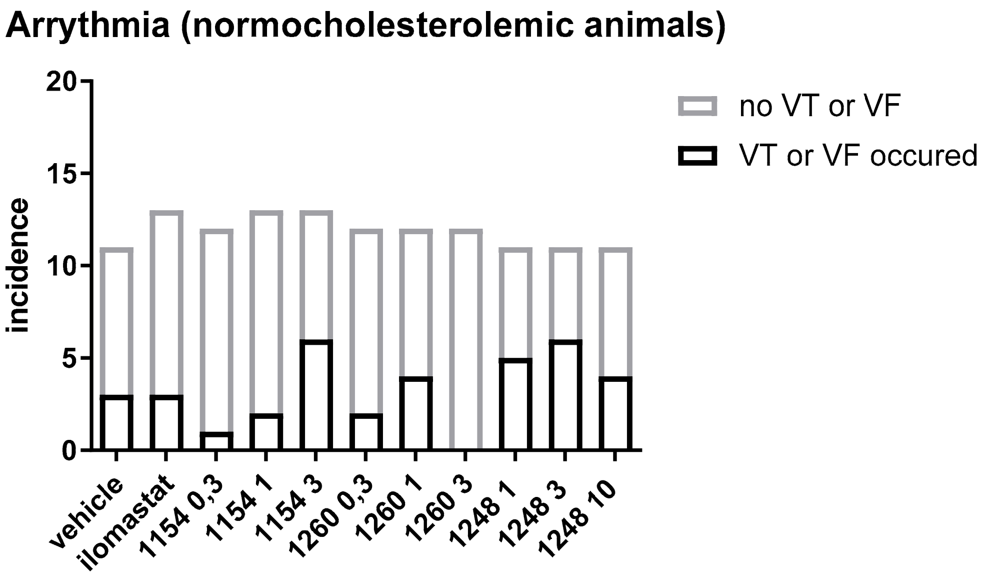

Supplementary Figure S5.Incidence of severe arrhythmias during 30min ischemia. LAD occlusion induced ischemia could trigger arrhythmia in the rat hearts. Test compounds as well as ilomastat or vehicle were given at 25th min of ischemia. Khi-square test, n=11-13, *p<0.05.

Comorbid animal model

Lipid panel

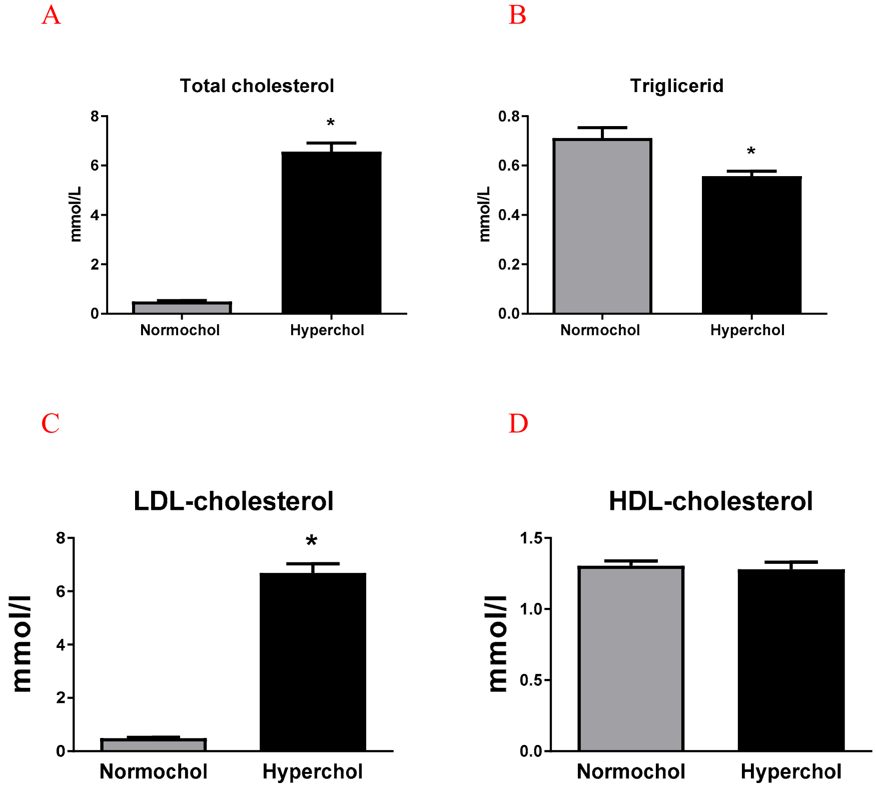

Supplementary Figure S6. Serum lipid panel of normocholesterolemic and hypercholesterolemic groups. After 12 weeks of diet, animals had a baseline blood sampling to validate the development of metabolic disease. LDL: low-density lipoprotein, HDL: high-density lipoprotein, IDL: intermediate-density lipoprotein. Student's t-test, n=60, *p<0.05.

Blood glucose level

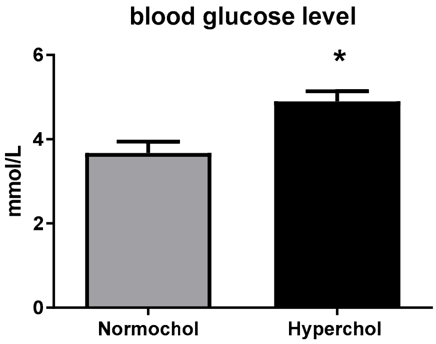

Supplementary Figure S7. Blood glucose level of normocholesterolemic and hypercholesterolemic groups. After 12 weeks of diet, animals had a baseline blood sampling to validate the development of metabolic disease. Student’s t-test, n=60, *p<0.05.

Body weight in normochol and hyperchol

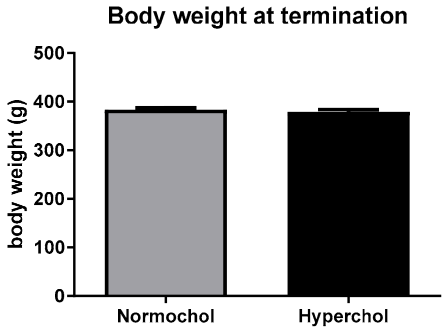

Supplementary Figure S8. Body weight of normocholesterolemic and hypercholesterolemic test groups. After 12 weeks of diet, animals had a baseline blood sampling to validate the development of metabolic disease. Student’s t-test, n=60, *p<0.05.

Mortality

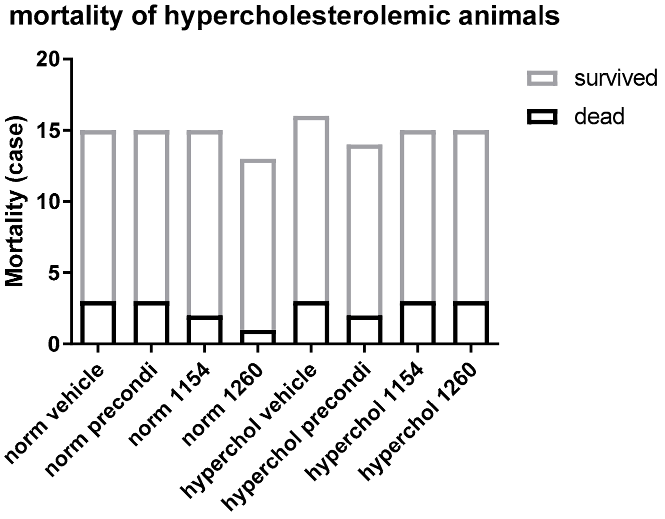

Supplementary Figure S9.All-cause mortality of normocholesterolemic and hypercholesterolemic comorbid model. Khi-square test, n=14-16, *$p<0.05$, no significant difference between groups.

Area at risk

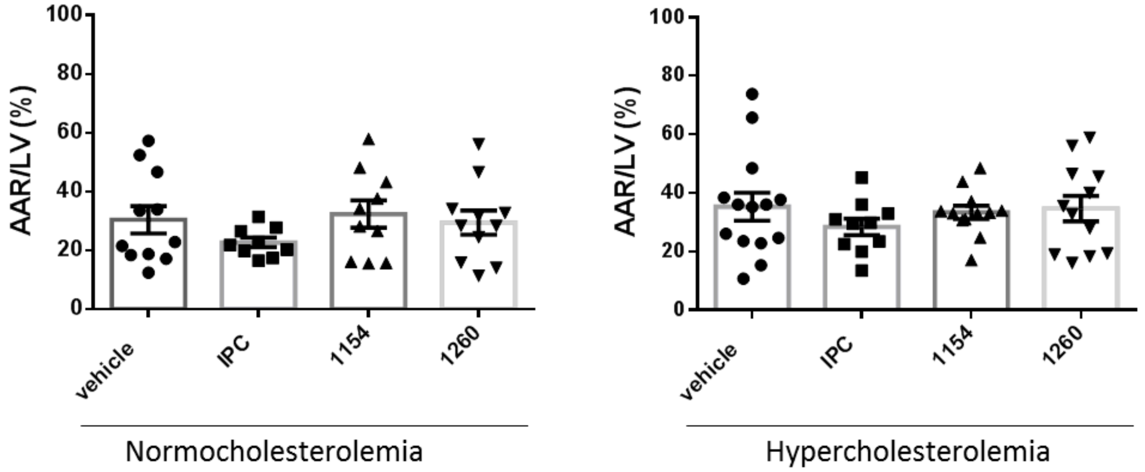

Supplementary Figure S10. Area at risk for normocholesterolemic and hypercholesterolemic test groups. AAR: area at risk, LV: left ventricle. One-way ANOVA, Fisher LSD post hoc test, n=10-12, *p<0.05.

Arrythmia

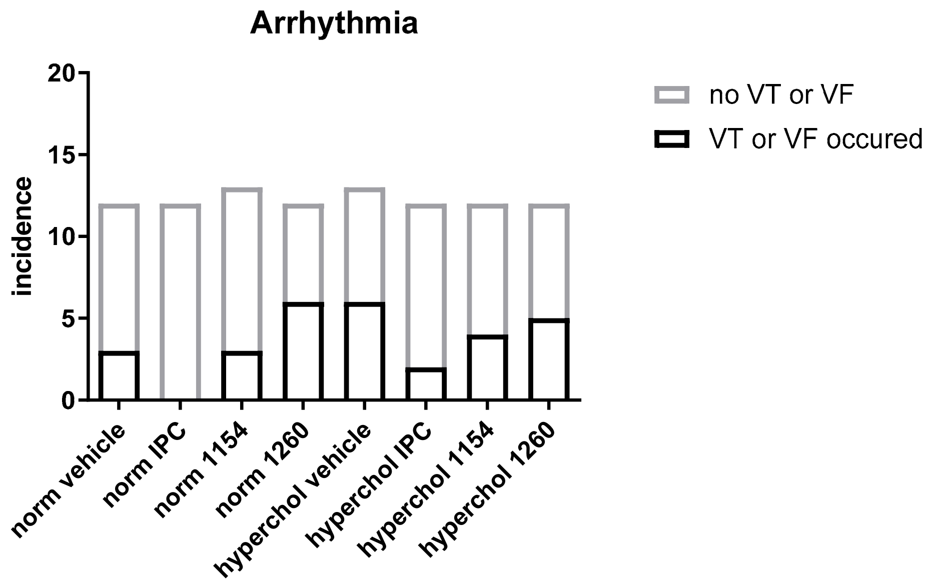

Supplementary Figure S11. Incidence of severe arrhythmias in hypercholesterolemic and age-matched normocholesterolemic test groups with positive control (IPC: ischemic preconditioning) and vehicle. Khi-square test, n=14-16, *p<0.05, no significant difference between groups.

Mean arterial blood pressure (MABP) and Heart rate of MMPI-1154 (normocholesterolemic)

MABP *Heart Rate*

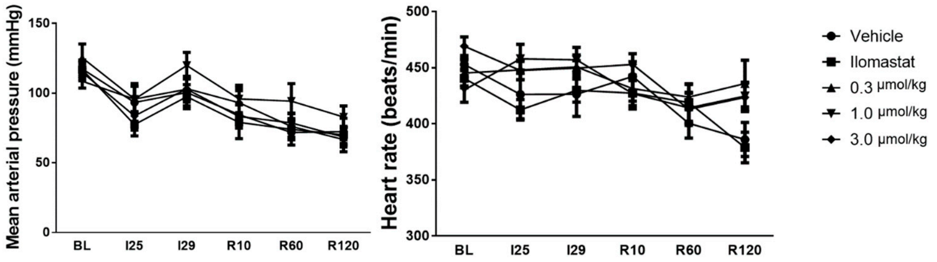

Supplementary Figure S12.A) Mean arterial blood pressure (MABP) and Heart Rate for MMPI-1154 test groups with Ilomastat and vehicle. BL: baseline, I: ischemia, R: reperfusion. Repeated-measures two-way ANOVA, n=11-15, *p<0.05, no significant difference.

Mean arterial blood pressure (MABP) and Heart rate of MMPI-1260 (normocholesterolemic)

MABP *Heart Rate*

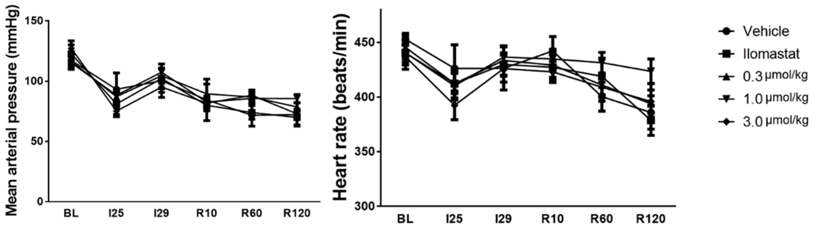

Supplementary Figure S12.B) Mean arterial blood pressure (MABP) and Heart Rate for MMPI-1260 test groups with Ilomastat and vehicle. BL: baseline, I: ischemia, R: reperfusion. Repeated-measures two-way ANOVA, n=11-15, *p<0.05, no significant difference.

Mean arterial blood pressure (MABP) and Heart rate of MMPI-1248 (normocholesterolemic)

MABP *Heart Rate*

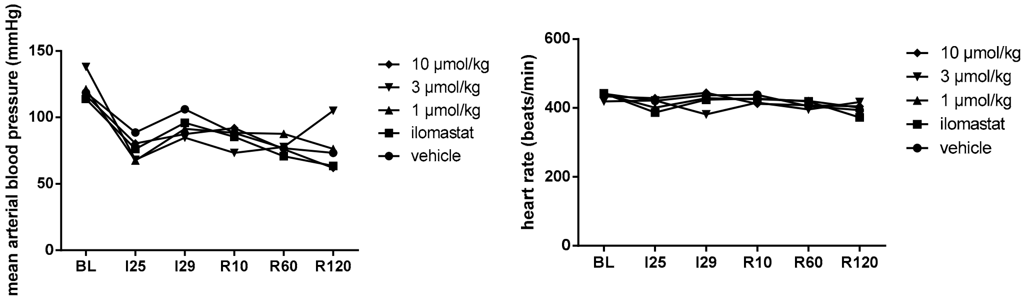

Supplementary Figure S12.C) Mean arterial blood pressure (MABP) and Heart Rate for MMPI-1248 test groups with Ilomastat and vehicle. BL: baseline, I: ischemia, R: reperfusion. Repeated-measures two-way ANOVA, n=11-15, *p<0.05, no significant difference.

Mean arterial blood pressure (MABP) in hypercholesterolemic and age-matched normocholesterolemic animals

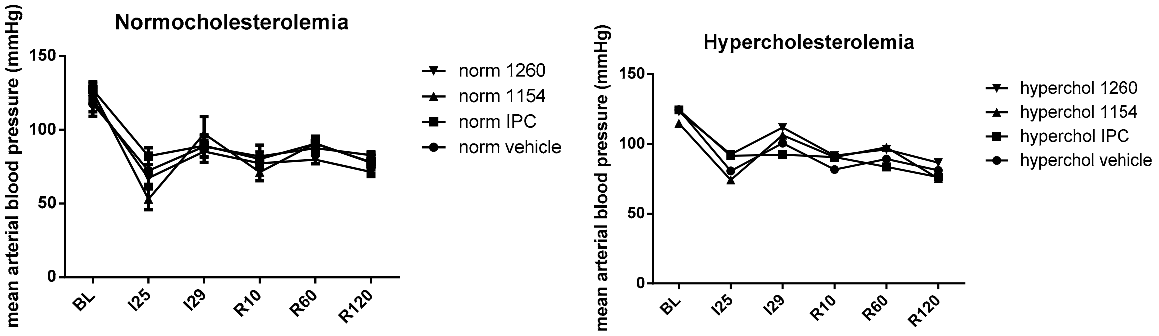

Supplementary Figure S13. Mean arterial blood pressure (MABP) of normocholesterolemic and hypercholesterolemic groups with positive control (IPC: ischemic preconditioning) and vehicle. BL: baseline, I: ischemia, R: reperfusion. Repeated-measures two-way ANOVA, n=11-15, *p<0.05, no significant difference in treatment.

Heart rate in hypercholesterolemic and age-matched normocholesterolemic animals

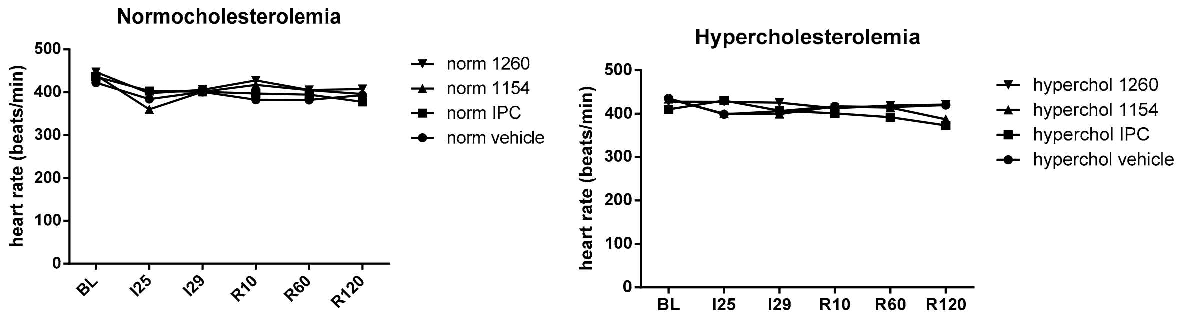

Supplementary Figure S14. Heart Rate of normocholesterolemic and hypercholesterolemic groups with positive control (IPC, ischemic preconditioning) and vehicle. BL: baseline, I: ischemia, R: reperfusion. Repeated-measures two-way ANOVA, n=11-15, *p<0.05, no significant difference in treatment.

Area at risk in vehicle groups

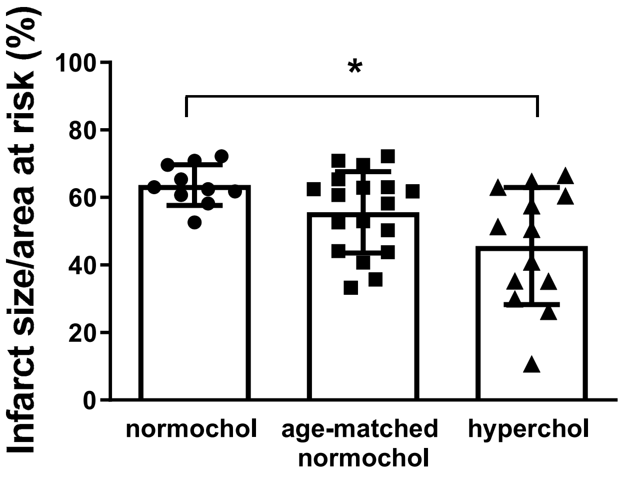

Supplementary Figure S15. Area at risk of vehicle-treated groups. One-way ANOVA Fisher LSD *post hoc* test, n=10-18, *p<0.05.

Gelatin zymography

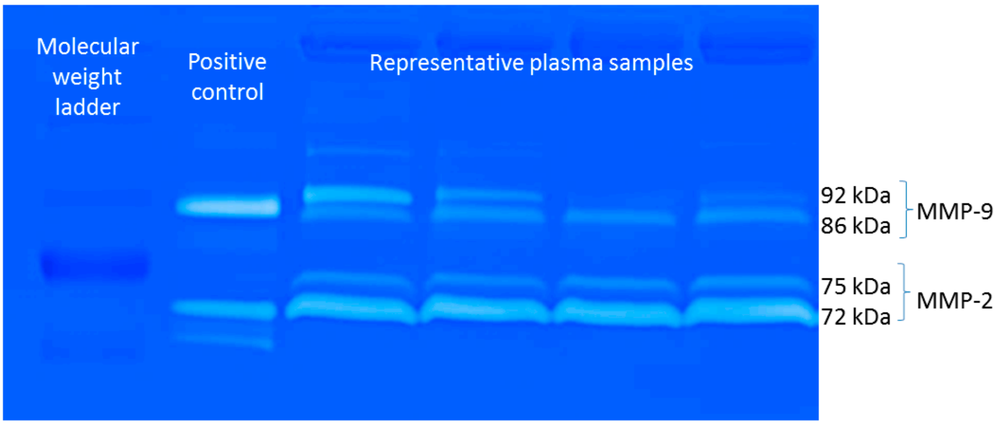

Supplementary Figure S16. Representative image of gelatin zymography. After electrophoresis and renaturation of the proteins, plasma samples were incubated at 37°C for 20 h in zymography development buffer, which enabled the active isoforms of matrix metalloproteinases (MMPs; 72 and 75 kDa for MMP-2 and 86 and 92 kDa for MMP-9) to degrade the gelatin content of the gel. Coomassie Brilliant Blue staining provided the dark blue background due to coloring of the co-polymerized gelatin in the gel, where the pale areas show bands for digested gelatin which size and intensity are proportional with the activity of MMPs.
